# Effectiveness and cost-effectiveness of a self-management training for patients with chronic and treatment resistant anxiety or depressive disorders: design of a multicenter randomized controlled trial

**DOI:** 10.1186/s12888-016-0927-1

**Published:** 2016-07-07

**Authors:** Maringa H. H. Zoun, Bauke Koekkoek, Henny Sinnema, Anna D. T. Muntingh, Anton J. L. M. van Balkom, Aart H. Schene, Filip Smit, Jan Spijker

**Affiliations:** Behavioural Science Institute, Radboud University Medical Center, PO Box 9104, 6500 HE Nijmegen, The Netherlands; Pro Persona Mental Health Care, Wolfheze 2, 6874 BE Wolfheze, The Netherlands; Research Group for Social Psychiatry & Mental Health Nursing, HAN University of Applied Science, PO Box 6960, 6503 GL Nijmegen, The Netherlands; Trimbos Institute (Netherlands Institute of Mental Health and Addiction), PO Box 725, 3500 AS Utrecht, The Netherlands; Department of Psychiatry and EMGO+ Institute, VU University Medical Centre and GGZ inGeest, A.J. Ernststraat 1187, 1081 HL Amsterdam, The Netherlands; Department of Psychiatry, Radboud University Medical Center, PO Box 9101, 6500 HB Nijmegen, The Netherlands; Department of Clinical, Neuro and Developmental Psychology, VU University Medical Centre, Van der Boechorststraat 1, 1081 BT Amsterdam, The Netherlands; Department of Epidemiology and Biostatistics, VU University Medical Centre, Van der Boechorststraat 1, 1081 BT Amsterdam, The Netherlands

**Keywords:** Anxiety, Depression, Chronic, Treatment resistant, Self-management, Randomized controlled trial, Specialized outpatient mental health care, Primary care

## Abstract

**Background:**

Many patients with anxiety or depressive disorders achieve no remission of their symptoms after evidence-based treatment algorithms. They develop a chronic course of the disorder. Current care for these patients usually consists of long-term supportive contacts with a community psychiatric nurse and pharmacological management by a psychiatrist. Data on the effectiveness of these treatments is lacking. A psychosocial rehabilitation approach, where self-management is an increasingly important part, could be more suitable. It focuses on the restoration of functioning and enhancement of patients’ autonomy and responsibility. Treatment with this focus, followed by referral to primary care, may be more (cost-)effective.

**Methods:**

A multicenter randomized controlled trial is designed for twelve participating specialized outpatient mental health services in the Netherlands. Patients with chronic and treatment resistant anxiety or depressive disorders, currently receiving supportive care in specialized outpatient mental health care, are asked to participate. After inclusion, patients receive the baseline questionnaire and are randomized to the intervention group or the usual care control group. The intervention focuses on rehabilitation and self-management and is provided by a trained community psychiatric nurse, followed by referral to primary care. Measurements take place at 6, 12, and 18 months after baseline. This study evaluates both the effectiveness (on quality of life, symptom severity, and empowerment), and cost-effectiveness of the intervention compared to usual care. In addition, a questionnaire is designed to get insight in which self-management strategies patients use to manage their disorder, and in the experiences of patients with the change of care setting.

**Discussion:**

In this study we evaluate the effectiveness and cost-effectiveness of a self-management intervention for patients with chronic and treatment resistant anxiety or depressive disorders in specialized outpatient mental health care. The results of this study may provide a first ‘proof-of-concept’ in this under-researched but important field, and might be relevant for a large group of patients in the context of a transition of the Dutch health care system.

**Trial registration:**

Netherlands Trial Register: NTR3335, registered 7 March 2012.

## Background

Anxiety and depressive disorders are among the most common mental disorders both worldwide and in the Netherlands. International data (the Netherlands and other developed countries) of the lifetime prevalence of these disorders are similar to each other. According to results from NEMESIS-2 (Netherlands Mental Health Survey and Incidence Study-2), the lifetime prevalence of anxiety disorders is 19.6 %, and 18.7 % for major depressive disorder [1, 2]. Also in specialized outpatient mental health care, anxiety and depressive disorders are common: 22.2 % of outpatients have an anxiety disorder and 37.2 % a depressive disorder [2].

A substantial part of patients in specialized outpatient mental health care achieve no remission of their symptoms after treatment that is provided in accordance with the (inter-)national multidisciplinary guidelines [3–5]. Their disorder seems treatment-resistant [6] and they suffer from a chronic course, with an estimated 41.9 % chronicity for anxiety disorders and 24.5 % for depressive disorders [7]. While anxiety disorders may be considered as chronic disorders in itself [8], a depressive disorder is defined as chronic when a depressive episode lasts more than two years [9]. The comorbidity between anxiety and depressive disorders is high (33–54 %) [10–12] and increases with a longer duration of symptoms. Comorbidity, in turn, increases the risk of treatment resistance and chronicity [13, 14]. Chronic and treatment resistant anxiety or depressive disorders are associated with more intense suffering, increased risk of suicide, and poorer social functioning [15, 16].

Care as usual (CAU) for patients with chronic and treatment resistant anxiety or depressive disorders usually consists of long-term supportive contacts with a key clinician, which is regularly a community psychiatric nurse, and pharmacological management by a psychiatrist. This CAU often has no clear purpose, and lacks treatment evaluation. Data on the effectiveness is lacking [17]. Nevertheless, termination of treatment is not considered to be an option due to the persistent symptoms, the level of suffering, and the absence of other treatment options. A psychosocial rehabilitation approach, focused on specific goals, could be more suitable [18, 19]. However, there is no research on feasibility and effectiveness of rehabilitation approaches for anxiety and depressive disorders in specialized outpatient mental health care.

In the development of such an approach for patients with chronic and treatment resistant anxiety or depressive disorders, self-management is an increasingly important element. Self-management for long-term health problems focuses on the restoration of functioning and enhancement of patients’ autonomy and responsibility. Self-management strategies are for instance: symptom management, enacting problem-solving strategies, and implementing learning plans [20]. We do not yet know much about the self-management strategies used by patients with chronic and treatment resistant anxiety or depressive disorders.

For patients with chronic and treatment resistant anxiety or depressive disorders in specialized outpatient mental health care the intervention, ZemCAD (English SemCAD; Self-management for Chronic Anxiety and Depression) was developed, in order to offer them a different perspective. This is also consistent with the transition of the Dutch health care system where the aim is to refer chronic patients to less intensive levels of care when possible. The focus in this study is on the transition from specialized outpatient mental health care to primary care. ZemCAD, the experimental treatment, offers treatment focused on rehabilitation and self-management, provided by a trained professional (a community psychiatric nurse, and in some cases a psychologist), followed by referral to primary care. Liaison consultation with specialized mental health care is an option if required. We compare the intervention to CAU.

The objectives of this study are:

1. To assess the cost-effectiveness of ZemCAD compared to CAU
2. To evaluate the effectiveness of ZemCAD compared to CAU with regard to quality of life, symptom severity, and empowerment
3. To get insight in the self-management strategies that patients use to manage their anxiety or depressive disorder;
4. To review the experiences of patients with a change of care setting.

Our hypothesis is that ZemCAD is more cost-effective than CAU. We expect that ZemCAD has outcomes just as good and not inferior to CAU with regard to quality of life, symptom reduction, and empowerment.

## Methods/Design

### Design

In a multicenter randomized controlled trial, with randomization at the level of patients in two parallel groups, the protocolized ZemCAD intervention versus care as usual is studied across twelve participating specialized outpatient mental health care services in the Netherlands. Assessments are at baseline, 6, 12 and 18 months and assessors are blind to randomization status.

### Setting/Participants

The twelve participating specialized outpatient mental health care services offer outpatient treatment interventions for anxiety and depressive disorders, in accordance with the (inter)national multidisciplinary guidelines. For patients with chronic and treatment resistant anxiety or depressive disorders, prolonged treatment (supportive contacts with a community psychiatric nurse) is offered. The transition from specialized outpatient mental health care tot primary care is not considered to be an option due to the persistent symptoms.

Inclusion criteria for the study are:

- ▪ patients with anxiety or depressive disorders according to DSM-IV;
- ▪ >18 years;
- ▪ > two years in specialized mental health care;
- ▪ received at least one psychological treatment and at least three medication steps according to the national multidisciplinary guidelines on anxiety and depressive disorders [21];
- ▪ regarded by their clinicians as treatment resistant, meaning that prolonged treatment in a specialized outpatient mental health service according to the professional is unlikely to improve clinical outcomes (e.g. achieve remission);
- ▪ currently having supportive contacts with a community psychiatric nurse.

Exclusion criteria are:

- ▪ life-threatening medical condition;
- ▪ dementia;
- ▪ psychotic disorder;
- ▪ bipolar disorder;
- ▪ alcohol or drugs dependence;
- ▪ not fluent in Dutch language;
- ▪ cognitive problems/indications for low IQ < 80.

### Recruitment and randomization

**Fig. 1:**
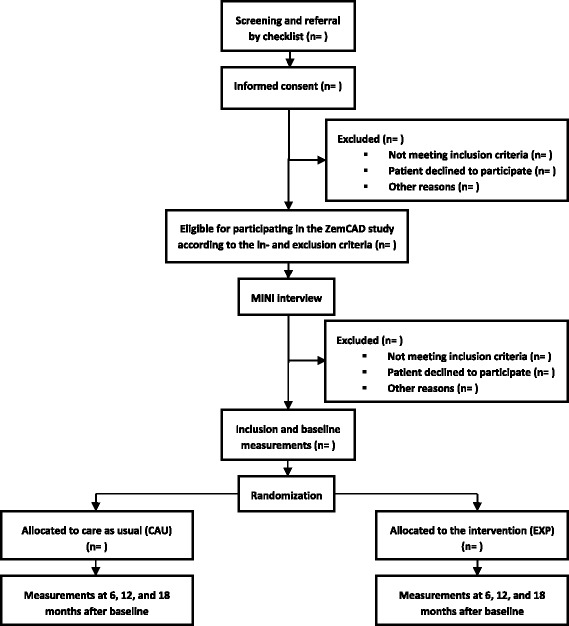
Study design and patient flow

Patients that are eligible for participating in the ZemCAD study according to the inclusion and exclusion criteria are asked to participate by their community psychiatric nurse. This nurse informs the patient about the study and provides an information letter to take home. If the patient is interested and consents to participate, the signed informed consent is sent to the coordinating research centre. To check the inclusion and exclusion criteria formally, the current DSM-IV disorders present in the patient were diagnosed with the Mini-International Neuropsychiatric Interview (MINI interview) in the mental health service that provides the treatment of the patient. The MINI is a well-validated semi-structured diagnostic interview used to establish psychiatric disorders according to the DSM-IV [22]. After inclusion, patients receive the baseline questionnaire. Patients and community psychiatric nurses are blind to the patient’s condition until they have completed the baseline questionnaire. Then the patient is allocated to ZemCAD or CAU, using a randomization schedule designed by an independent statistician. To evenly distribute ZemCAD/CAU across community psychiatric nurses, block randomization (block size of four) of patients is used. Questionnaires are completed over the internet. If a patient has no access to the internet, the questionnaires are completed on paper. Patients are informed that participation in the study is voluntary and that they can withdraw from the study at any time. An overview of the study design and patient flow is provided in Fig. 1.

### Intervention

ZemCAD is based on an existing treatment protocol for patients with chronic depression, developed at the Amsterdam Academic Medical Center [23], adapted for patients with chronic and treatment resistant anxiety or depressive disorders [24]. During thirteen sessions over twenty-six weeks patients and their families are educated about the nature of their chronic disorder, about suicidality and crises, and they learn how to cope with these conditions and events. The trained professional and the patient make an action plan to re-establish social contacts and improve their daily living activities.

ZemCAD consists of three parts. The first part is an introduction phase of three weeks with weekly sessions. The goals for the professionals are to get acquainted with the patient and family, and explain the treatment. Patients make an individual treatment plan, identify symptoms and daily activities, keep a log of symptoms, and learn how they can accept lifestyle changes due to having a chronic disease. The second part is a coaching and treatment phase of fourteen weeks with sessions every second week. Patients start again engaging in social activities, they are stimulated to maintain a daily structure, and learn to use general problem solving skills to cope with their chronic disease. The third part is the final phase of nine weeks with sessions every three weeks. Topics are to make an action plan on how to deal with crisis situations and to further practice with earlier mentioned skills. Finally, patients are referred to primary care. Every primary care practice is asked to select a mental health professional who works in close collaboration with the general practitioner and actively monitors functioning of the patient. After referral to primary care, the general practitioner is responsible for the prescription of medication. Both mental health professional in primary care and general practitioner have easy access to specialized outpatient mental health services for liaison consultation if required.

Patients receiving ZemCAD are allocated to a new and trained professional. This clearly marks the transition from CAU to ZemCAD for both patient and professional. The training of the participating professionals in ZemCAD consists of a two-day course. A prerequisite is that the professionals study the intervention in advance. The two-day course is provided by an expert and trainer in cognitive behavioural therapy and motivational skills for anxiety and depressive disorders. The training combines self-study, lectures, assignments, and group discussions. During the study, three additional booster sessions were given. In addition, in each mental health service monthly booster sessions for the professionals were scheduled.

#### Usual care

Patients who are allocated to CAU continue to receive specialized outpatient mental health care, which usually consists of long-term supportive contacts with a community psychiatric nurse, and pharmacological management by a psychiatrist. In most cases patients continue care with their own community psychiatric nurse. Only if the patient allocated to CAU receives treatment from a community psychiatric nurse who is trained to provide the intervention, the patient needs to switch to a different (untrained) community psychiatric nurse. CAU may also involve termination of treatment in specialized outpatient mental health care and referral to primary care. In the ZemCAD intervention, however, this referral is planned and anticipated within the intervention, while in CAU the referral and termination of the treatment only occurs when indicated.

### Treatment integrity

Patients who are allocated to CAU in most cases continue care with their current community psychiatric nurse. Since there is no clear treatment guideline for CAU, treatment integrity is not assessed in the control group. We do, however, monitor the number of contacts patients have with health care professionals. In the intervention group, treatment integrity is assessed for each participating patient using a checklist that the trained professional completes at the end of each treatment session. In this checklist the trained professional can indicate on which points the treatment differs from the ZemCAD protocol.

### Measurements

At baseline demographics are measured. Measurements take place 6, 12, and 18 months after baseline. Quality of life is measured with the World Health Organization Quality of Life instrument, Brief version (WHOQOL-BREF) [25, 26]. The WHOQOL-BREF measures the overall quality of life in four different areas: physical health, psychological health, social relationships and environment. Anxiety symptoms are measured with the Beck Anxiety Inventory (BAI) [27], a validated self-rated questionnaire to assess the severity of anxiety symptoms. Depression severity is measured with the Patient Health Questionnaire (PHQ-9) [28, 29], a validated self-rated questionnaire to assess the severity of depression symptoms. Empowerment is assessed using the Dutch Empowerment Scale (Nederlandse Empowerment Vragenlijst) [30]. Contacts with health care professionals, medical costs, and productivity costs are measured with the Trimbos and Institute of Medical Technology Assessment questionnaire for costs associated with psychiatric illness (TiC-P) [31]. Using this self-completion questionnaire, direct and indirect costs of health care utilization of patients can be assessed. In addition, the EuroQoL (EQ-5D) [32] is used to compute Quality Adjusted Life Years (QALYs) gained, to be used in the cost-utility analysis.

To get insight in self-management strategies used by patients with chronic and treatment resistant anxiety or depressive disorders, and experiences of patients with a change of care setting, a questionnaire is designed. We do this by organizing focus groups, and use concept mapping [33] for processing the data. The questionnaire is administered during the ZemCAD trial, to all participants, in both conditions. An overview of measurements is provided in Table 1.

**Table 1:** Overview of measurements

	Baseline	6 months	12 months	18 months
Demographics	x			
Clinical depression/anxiety (MINI)	x			
Quality of life (WHOQOL-BREF)	x	x	x	x
Anxiety (BAI)	x	x	x	x
Depression (PHQ-9)	x	x	x	x
Empowerment (Dutch Empowerment Scale)	x	x	x	x
Health care costs (TiC-P)	x	x	x	x
Health care costs (EQ-5D)	x	x	x	x
Questionnaire about self-management strategies and experiences with a change of care setting				x

### Data handling

Each participant receives a code. This code is a unique number that is not based on personal information. The list linking participant code and personal information is kept in a separate file stored in a database with access limited to designated researchers and data managers. All our study-related information is stored in secure folders with limited access. Paper-based data collection forms only contain participant codes to maintain participant confidentiality and are stored in a locked cabinet in an area with limited access. Electronic data files are stored on a file system with access restricted to designated researchers and data managers. All results will be described in groups of participants and data will not be traced to a single person. Data will be stored for 5 years after the end of inclusion. The study has low to negligibly risks therefore no data monitoring committee is assigned. Only the designated researchers, or persons assigned by them, will have access to the final data set. Authors of the final manuscript have made substantial contributions to the design, conduction, interpretation and reporting of the trial. We will not use professional writers. Participants, funders and involved professionals will receive a summary of the study results. Final results will be presented via publications and presentations.

### Sample size

The feasibility of our trial is evaluated in a pilot study, which was carried out within one of the participating mental health services. Based on the number of included patients in this pilot and the number of services participating, we expect to be able to include 130–180 patients in our trial. This is enough to detect a standardized mean difference of d ≥ 0.50 in a 2-tailed test at *α* = 0.05 and with a power of (1-β) = 0.80. However, we do not expect such large differences between the conditions on any of the outcomes. With regard to the hypotheses about the clinical effects we assume non-inferiority rather than superiority and with *n* = 130 the non-inferiority margin would be as large as *d* = 0.45 (one sided), which is uninformative. That said, our main hypothesis requires health-economic decision-making. In that context it is more customary to take a probabilistic medical decision-making approach and do not rely on statistical techniques to test hypotheses.

### Statistical analysis

Descriptive statistics are used for presenting the demographics of the sample. All outcome analyses are conducted in agreement with the intention-to-treat (ITT) principle, as required by the CONSORT statement [34]. To that end, we use linear mixed-effects models. If differences in baseline data are present, we adjust for relevant baseline characteristics, by including baseline variables as covariates in the model. To see how effects develop over time we also model interactions of the treatment with time in the linear mixed-effects models. All analyses are conducted in SPSS 22 and Stata 13.1 or later versions.

### Economic evaluation

The health economic evaluation is conducted in agreement with the CHEERS statement [35]. Healthcare utilization is measured and costs are calculated using standard unit prices (for the reference year 2014) published in the Dutch costing guideline [36]. In addition, data on (increased or decreased) productivity due to changes in the number of hours worked per week and accounting for absenteeism and presenteeism are valued using the mean per-capita gross income. The difference between ZemCAD and CAU in terms of health gains are expressed in QALYs, based on the EQ-5D. Non-parametric bootstrapping is used to estimate the uncertainty surrounding costs and effects. On that basis, probabilistic statements can be made about the likelihood that, relative to usual care, the new intervention (1) costs more and is associated with QALY gains, (2) costs more and is associated with QALY losses, (3) costs less and is associated with QALY losses, or (4) costs less and is associated with QALY gains. Obviously scenario 2 is the least attractive and in that case maintaining the old (current) health care system is the best option as seen from a (narrow) health-economics perspective, but always contingent on further medical-ethical considerations. In case of scenario 4 the new health care system is the best option as viewed from a health-economics perspective. However, we often end up in scenario 1: we pay more and get more QALYs. This could be an acceptable scenario, but raises the question how much we are willing to pay for the additional QALY gains. In such a scenario we therefore also consider a range of willingness-to-pay (WTP) ceilings while using the so-called acceptability curve, where the probability of cost-effectiveness is plotted against various WTP ceilings. The incremental cost-effectiveness ratios (ICER) acceptability curve helps in the decision whether ZemCAD should or should not be considered as cost-effective from a probabilistic medical decision-making perspective. The health-economic evaluation is completed with sensitivity analyses focusing on uncertainties in major cost-drivers, and is intended to show how robust the results of the cost-effectiveness analysis are under uncertainty. It is worth re-emphasising that in addition to health-economic considerations other considerations, such as medical-ethical considerations and patients’ preferences, ought to play a role in medical decision-making.

## Discussion

This article describes a protocol for a multicenter randomized controlled trial on the effectiveness and cost-effectiveness of a self-management intervention for patients with chronic and treatment resistant anxiety or depressive disorders in specialized outpatient mental health care. In this study we evaluate the cost-effectiveness of ZemCAD compared to CAU. We also explore the effects of the ZemCAD intervention compared to CAU on quality of life, depressive and anxiety symptom severity, and empowerment. Furthermore we like to shed light on the self-management strategies that patients use to cope with their chronic and treatment resistant anxiety or depressive disorder, and experiences of patients with a change of care setting. Given the fact that there are hardly any evidence-based interventions for patients with these disorders, and the need to make cost-effective use of scarce resources, the results of this study may provide a first ‘proof-of-concept’ in this under-researched but important field. Moreover the results of this study might be relevant for a large group of patients in the context of a transition of the Dutch health care system. In this study we focus on the transition from specialized outpatient mental health care to primary care.

## Abbreviations

BAI, Beck Anxiety Inventory; CAU, care as usual; EQ-5D, EuroQoL; ICER, incremental cost-effectiveness ratio; ITT, intention-to-treat; MINI interview, Mini-International Neuropsychiatric Interview; NEMESIS-2, Netherlands Mental Health Survey and Incidence Study-2; PHQ-9, Patient Health Questionnaire; QALYs, Quality Adjusted Life Years; TiC-P, Trimbos/iMTA questionnaire for Costs associated with Psychiatric illness; WHOQOL-BREF, World Health Organization Quality of Life instrument Brief version; WTP, willingness-to-pay; ZemCAD (English SemCAD), Self-management for Chronic Anxiety and Depression
